# Pseudo-CT generation in brain MR-PET attenuation correction: comparison of several multi-atlas methods

**DOI:** 10.1186/2197-7364-2-S1-A29

**Published:** 2015-05-18

**Authors:** Ines Merida, Nicolas Costes, Rolf Heckemann, Alexander Hammers

**Affiliations:** Université de Lyon 1, INSERM, CNRS, Lyon Neuroscience Research Center, Lyon, France; LILI-EQUIPEX – Lyon Integrated Life Imaging: hybrid MR-PET, Lyon, France; Siemens Healthcare France SAS, Saint-Denis, Paris, France; CERMEP-Imagerie du vivant, Lyon, France; MedTech West at Sahlgrenska University Hospital, Gothenburg, Sweden; Neurodis Foundation Lyon, Lyon, France

Simultaneous MR-PET imaging opens new perspectives for understanding many aspects of brain function. However, accurate brain attenuation correction (AC) is required for absolute PET quantification. Radiodensity maps for AC are not readily available in PET-MR. Various strategies have been proposed to generate substitute attenuation maps. Recently, it has been shown that multiatlas techniques for AC outperform methods based on MR imaging sequences, such as Ultrashort-Echo-Time. We recently investigated several multiatlas methods to build a patient-specific pseudoCT, all based on multiatlas registration and label propagation, and introduced a novel maximum probability method (MaxProb). Here, a new database of 40 MR/CT image pairs (atlases) was used. Pairwise nonrigid registration of each atlas MR image to the target MR image yielded a geometric transformation that we used for propagating the atlas CT into the target’s space. The synthetic CT was generated by voxelwise atlas selection and intensity averaging. We implemented a state-of-the-art algorithm along with two variants and MaxProb. A pseudoCT was generated for each subject of the database and assessed by comparison with the real CT in a leave-one-out scheme. Jaccard overlap indices were computed per tissue class (air, soft tissue, and bone). Voxel classification error was also assessed. The Jaccard indices and voxel classification error showed that MaxProb (8.31±1.12%) performed slightly better than Ref (8.65±0.93%). The influence of the number of atlases fused in pseudoCT generation was studied, showing that an optimal number improves accuracy. By adjusting the number of fused atlases, it was possible to significantly improve on Ref (8.46±0.96%). The methods now need evaluation on quantitative PET data to identify potential benefits in PET analysis.

